# The Challenge of ICIs Resistance in Solid Tumours: Could Microbiota and Its Diversity Be Our Secret Weapon?

**DOI:** 10.3389/fimmu.2021.704942

**Published:** 2021-08-20

**Authors:** Michela Roberto, Catia Carconi, Micaela Cerreti, Francesca Matilde Schipilliti, Andrea Botticelli, Federica Mazzuca, Paolo Marchetti

**Affiliations:** ^1^Department of Clinical and Molecular Medicine, Sant’ Andrea University Hospital, Sapienza University of Rome, Rome, Italy; ^2^Medical Oncology Unit, Policlinico Umberto I, Sapienza University of Rome, Rome, Italy; ^3^Department of Clinical and Molecular Medicine, Faculty of Medicine and Psychology, Sant’ Andrea University Hospital, Sapienza University of Rome, Rome, Italy

**Keywords:** microbiota, immunotherapy, immune checkpoint inhibitors (ICIs), fecal microbiota transplantation (FMT), diet, nutrients

## Abstract

The human microbiota and its functional interaction with the human body were recently returned to the spotlight of the scientific community. In light of the extensive implementation of newer and increasingly precise genome sequencing technologies, bioinformatics, and culturomic, we now have an extraordinary ability to study the microorganisms that live within the human body. Most of the recent studies only focused on the interaction between the intestinal microbiota and one other factor. Considering the complexity of gut microbiota and its role in the pathogenesis of numerous cancers, our aim was to investigate how microbiota is affected by intestinal microenvironment and how microenvironment alterations may influence the response to immune checkpoint inhibitors (ICIs). In this context, we show how diet is emerging as a fundamental determinant of microbiota’s community structure and function. Particularly, we describe the role of certain dietary factors, as well as the use of probiotics, prebiotics, postbiotics, and antibiotics in modifying the human microbiota. The modulation of gut microbiota may be a secret weapon to potentiate the efficacy of immunotherapies. In addition, this review sheds new light on the possibility of administering fecal microbiota transplantation to modulate the gut microbiota in cancer treatment. These concepts and how these findings can be translated into the therapeutic response to cancer immunotherapies will be presented.

## Introduction

Over the past few decades, significant progress has been achieved in cancer treatment, with immunotherapy becoming a research hotspot in recent years (1). The last years have seen unprecedented clinical responses and rapid drug development, accumulating reports of advanced cancer patients defying the odds and achieving complete remissions with immunotherapy treatments (2).

Immunotherapy is a powerful strategy to treat cancer by harnessing the body’s immune system to generate or augment an immune response against it (3). This is accomplished by either training resident immune cells to recognize and eliminate cells bearing tumor specific antigens, providing external stimuli to enhance immune mediated tumor cell lysis or abrogating signals directed by tumor cells to dampen immune responsiveness (4). Both cellular and molecular components of the tumor microenvironment can affect the efficacy of immunotherapy (5).

The tumor microenvironment has been recognized as a key factor in tumor development and progression (6). Many of its components influence cancer cell malignant behavior, within its three-dimensional structure (1, 2). Non-malignant cells include immune cells, cells of the vasculature and lymphatic system, cancer-associated fibroblasts, pericytes, and adipocytes (7). The communication between cell types is driven by an extremely complex network of cytokines, chemokines, growth factors, other inflammatory mediators, and matrix remodeling enzymes (8).

The intestinal microbiota is the collection of all microorganisms (eukaryotes, bacteria, virus) living in human gastrointestinal tract. Microbiome may be very different between individuals, and it is constantly influenced by age, nutrition, antibiotic use, smoking, alcohol. There is a continuous interaction and interplay between microbiome and the immune system, and the microbiota seems to play a role in the pathogenesis of various inflammatory diseases such as NASH, inflammatory bowel disease and obesity (9).

The human microbiome has recently been described as a component of various tumor microenvironments, due to its ability to impair tumor cell metabolism by maintaining a healthy mucosal barrier, to induce inflammation, and to produce genotoxins and different bacterial metabolites (10). It has been estimated that the total number of bacteria in the 70 kg average human male is 3.8·10^13^ and that 10% of metabolites found in mammalian blood are derived from the gut microbiota (11, 12). Indeed, humans and their microbiome are considered to form a composite organism, a so-called holobiont, that defines humans together with their connected microbial network, instead of merely autonomous eukaryotic organisms (13, 14). Furthermore, a clear interplay between the local microbiome, the intestinal epithelium, and resident immune cells has recently begun to emerge, where all participants actively foster gastrointestinal homeostasis. In this system, bacterially derived metabolites serve as important signals that continuously contribute to the proper function of the epithelial barrier and immune cells (14).

Over the last decade, researchers have found a consistent connection between a dysfunctional gut microbiota (dysbiosis) and various cancers, such as cancers of the urinary tract, cervix, skin, airways, colon, breast, and lymphomas (10, 15). Considering that the primary characteristics of microbiota dysbiosis are alterations of bacterial species and the increase of pathogenic bacteria (16), studying the microbial communities in the tumor microenvironment may shed light on the role of host-bacteria interactions in cancer.

The relation between cancer and microbiota is also influenced by other factors. Out of the multiple host-endogenous and host-exogenous factors involved in the modulation of the composition of gut microbiota, such as diseases, drugs, and smoke (17), diet emerges as a pivotal determinant of its community structure and function (18). Considering that the populations of dominant species within the human colonic microbiota can potentially be modified by dietary intake to influence health (19), the responses of the gut microbiota to various factors are considered to be a valuable tool to exploit in order to develop new strategies to promote human health.

Therefore, it is important to identify gut resident bacteria. Metagenomics and culturomics are the tools used to study human microbiota, to understand and detect gut microbes, to identify their specific role in the microenvironment and correlate all data with clinical specifical situations (20, 21).

**Figure 1:**
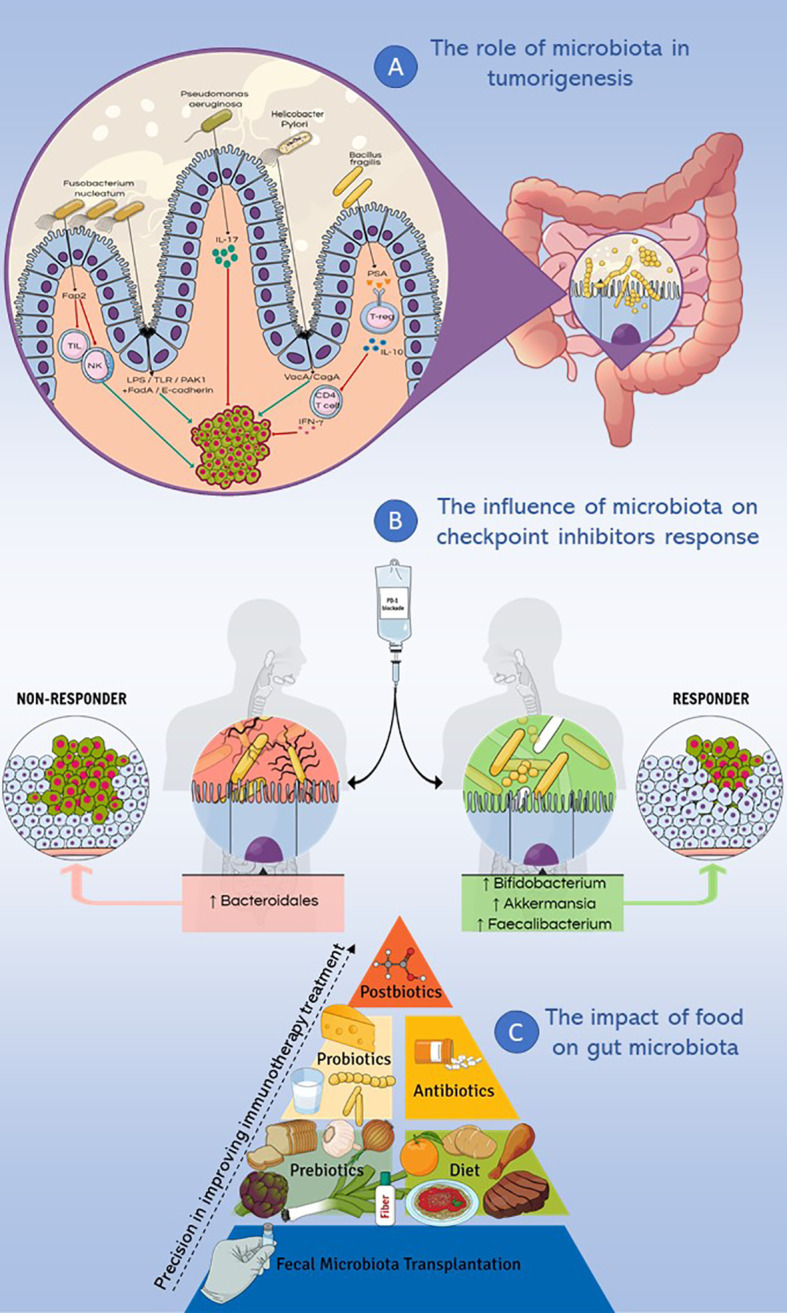
Microbiota and immunotherapy resistance. This figure summarizes the main topics discussed in the review. **(A)** Different genera such as *Fusobacterium nucleatum, Pseudomonas aeruginosa, Helicobacter Pylori* and *Bacillus fragilis* were studied for their implication in cancer pathogenesis, causing inflammatory and/or immune response, DNA damage and modulating cell proliferation. **(B)** Microbiota influences the response to checkpoint inhibitors therapy: the enrichment of fecal microbiota with *Akkermansia muciniphila, Faecalibacterium* spp and *Bifidobacterium* spp correlates with a positive response to PD-1 immune-checkpoint blockade, while a higher abundance of *Bacteroidales* correlates with a deficient response to the same treatment. **(C)** Different dietary nutrients modify the response to immunotherapy, ranging from fecal microbiota transplantation to the use of postbiotics, with increasingly precise effects on the treatment response.

Considering the increasing interest in the microbiota composition of oncological patients, the aim of this review is to analyze the role of microbiota in cancer promotion, its effects on the immune system and its emerging role as a response modulator to immunotherapy-based cancer treatments. In this perspective, this review focuses on understanding how the diet and the use of probiotics, prebiotics, postbiotics and antibiotics might modify the composition of the gut microbiota and, consequently, the therapeutic response to cancer immunotherapies (**Figure 1**).

## The Role of Microbiota in Tumorigenesis

Given the variability of gut microbiota between individuals due to external influences such as diet (22), host genetic background and other environmental factors, many studies employed both tumor and normal tissue samples taken from the same individual, in order to provide a more accurate view of the tumor-associated shifts in the microbiome (22, 23). The general conclusion is that tumor microenvironments harbor microbiomes distinct from those of normal tissue microenvironments. Various analyses consistently showed variation in the bacterial phyla abundance when comparing the matched normal and tumor tissues, demonstrating that there is indeed a cancer-associated signature in the tumor microbiome (24–26).

Gut microbiota can be divided into 3 clusters according to the effects of the microbes on the human body: beneficial, neutral, and pathogenic (27). The first group comprehends *Bifidobacterium* and *Lactobacillus*, which can protect the intestinal tract, produce beneficial metabolites, and detoxify the human gut. Neutral microbes, such as *Enterococcus*, have dual characteristics, being beneficial to human health in normal growth conditions and being able to cause different degrees of diseases when exceeding a certain standard growth or transferred to other parts of the body (28). Pathogenic microbes, such as *Salmonella* and *Helicobacter pylori*, secrete toxins and thus might cause disease (29).

The gut microbiota has differential effects on tumorigenesis, in fact bacteria may be tumour suppressive for cancer, especially at distal sites by releasing metabolites and immune modulators such as histone deacetylase (HDACi), hypoxia induced factor (HIF), interkeukin-10 (IL-10) that enrich gut barrier function and have an antioxidant effect (30). Moreover, it is important to consider the role of TME and the gut mucosal barrier: the increased permeability of gut mucosal barrier is correlated with inflammation and development of cancer. Literature data describes a link between integrity of gut mucosal barrier and differential faecal bacteria (31).

Lacking bacterial diversity in the intestine is the key feature for many intestinal and extraintestinal disorders. Considering the evident differences in the nutrient composition of the tumor microenvironment and the metabolic activity of microbiota, there is an unquestionable metabolic interaction between the tumor and its own microbiota (32). It is suggested that tumorigenesis is promoted by a combination of intestinal microbiota alterations (e.g., increased abundance of *Escherichia coli* and *Fusobacterium nucleatum*), rather than a difference in the abundance of a specific strain (33).

New evidence points to the association between the gut microbiota and the development and progression of gastrointestinal cancers such as colorectal cancer and hepatocellular carcinoma (34), as well as cancers of the respiratory system, where microbiota’s dysbiosis in heavy smokers, together with the epithelial integrity loss, could initiate inflammation in lung cancer (35). Moreover, the relationship between human microbiota and other types of cancers, such as breast cancer, is starting to emerge (36).

As an example of the role of microbiota in cancerogenesis, here it is described the hypothesis that emerged to explain the contribution of bacteria to colorectal cancer (CRC) carcinogenesis. On one hand, the presence of a dysbiotic microbial community with pro-carcinogenic features can remodel the microbiome towards pro-inflammatory responses and epithelial cell transformation, thus leading to cancer. On the other hand, the “driver-passenger” theory states that the so-called “bacteria drivers” could initiate CRC by inducing epithelial DNA damage leading to tumors with indigenous ability to promote the proliferation of “passenger bacteria”, by means of a growth advantage in the tumoral microenvironment (37, 38). These bacteria hardly colonize a healthy colon and cannot breach the intact colon wall, but they can easily invade a broken colon wall in the context of adenoma or carcinoma (37, 39). A highly diverse gut microbiota might be a key feature of a healthy gut, a balance between driver and passenger bacteria might create a species-rich ecosystem which is able to deal with environmental stresses that promote CRC (40).

Different studies aimed to identify potential “driver” bacteria. *Bradyrhizobium japonicum* was found to be increased in lung cancer patients with early-stage tumors (stages I and II) when compared to patients with advanced-stage tumors (III and IV) (41). Moreover, in patients with breast cancer, the analysis of 16S rRNA showed a higher relative abundance of *Bacillus* spp. compared with healthy samples, and *Methanobacteriaceae* was richer in malignant disease compared to benign disease (42, 43). The abundances of driver and passenger bacteria may serve as a primary indicator of cancer initiation risk and development.

### Suspected Role-Players in Carcinogenesis

The human gut microbiota is dominated by 3 primary phyla: *Firmicutes* (30%-50%), *Bacteroidetes* (20%-40%) and *Actinobacteria* (1% - 10%). Some strict anaerobes, as well as *Bacteroides*, *Eubacterium*, *Bifidobacterium*, *Fusobacterium*, *Peptostreptococcus* and *Atopobium* (44), constitute a major portion of the gut microbiota, while facultative anaerobes, such as *Lactobacilli*, *Enterococci*, *Streptococci* and *Enterobacteriaceae*, represent a minor proportion (45).

During their phylogenetic evolution, bacteria progressively acquired virulence factors that conferred pathogenicity. In this regard, bacteria developed the ability to penetrate the gut mucosal barrier, as well as the ability to adhere to and invade intestinal epithelial cells, using flagella, pili, and adhesins (46–48). These virulence factors are considered to be one of the elements that determine disease-promoting and pro-carcinogenic effects of pathogens (49).

Intestinal bacteria contribute to carcinogenesis in different ways, causing inflammatory and/or immune response, DNA damage and modulating cell proliferation. Different genera were studied to prove their implication in cancer pathogenesis, especially in CRC. A recent study showed how colorectal cancer samples were dominated by *Firmicutes*, *Bacteroidetes*, and *Proteobacteria* (22). Tumors showed an enrichment of *Proteobacteria* and a depletion of *Firmicutes* and *Bacteroidetes*, underlining the evident and significant changes in these phyla between the normal and cancer states. There was also an increase in the phylum *Fusobacteria* in the tumor-associated microbiome (22). The important findings were that two of the genera that have been found to be enriched in the tumor microbiome, *Providencia* and *Fusobacteria*, are already known to be pathogenic. Moreover, *Fusobacteria* has been implicated in CRC by many other studies (50, 51). The presence of species belonging to the genera *Providencia* and *Fusobacterium* in the tumor microenvironment may suggest that they could have a role in oncogenesis or tumor progression, or that the tumor’s niche favors them.

Several studies suggest that *Fusobacteria* is likely a cancer driver and its carcinogenic mechanism has been unveiled (52, 53). The discovery of *Providencia* in the tumor microbiome is interesting as it produces an immunogenic lipopolysaccharide that participates in epithelial barrier dysfunction and endothelial apoptosis (54). These factors generally lead to gastroenteritis, but its association with the tumor environment may suggest that it should be studied as a cancer-promoting pathogen. Interestingly, *Fusobacteria* and *Providencia* share many important phenotypic characteristics such as the ability to damage colorectal tissue and to encode several virulence genes that are responsible for lipopolysaccharide biosynthesis, which are also significantly increased in the tumor microenvironment (22).

In the same way, certain CRC-associated *Escherichia coli* strains acquired virulence factors, such as the afa and eae adhesins, which conferred the ability to adhere to and invade the intestinal epithelium (55, 56). *E. Coli* is indeed a common gut commensal bacterium, but it has been shown to be able to colonize the colonic mucosa; it increases mucosal permeability through the activation of Wnt mitogenic signaling, it damages the DNA and interferes with the DNA repair process, hence inducing CRC development (57).

Other common pathogenic bacteria have been studied for their association with carcinogenesis. A study showed that CRC patients and precancerous lesions had a higher expression level of *Salmonella* flagella antibodies than healthy controls, with diet differences being one of the mediating factors, suggesting a potential link between *Salmonella* and CRC (58). Furthermore, *Salmonella* can secrete the effector protein AvrA to promote acetylation and ubiquitination of target proteins. AvrA inhibits β-catenin degradation, maintains β-catenin stability, and promotes intestinal epithelial cell proliferation, thereby facilitating tumorigenesis, increasing tumor diversity, and driving tumor progression (59).

## The Influence of Microbiota on Checkpoint Inhibitors Response

It has recently been shown that gut microbiota influences the host immune response to different cancer therapies, such as radiotherapy, chemotherapy, stem cell transplant and immunotherapy, by upsetting drug metabolism, the anti-tumor effects and the toxicity of the medications currently used (60).

ICIs immunotherapy is based on using natural and artificial components in order to promote or induce the natural immune system to neutralize cancer cells (61, 62). Since the introduction of ICIs, there has been a change in the treatment of advanced cancer by introducing immunotherapy as a recognized first and second-line therapies. ICIs are monoclonal antibodies which target inhibitory receptors on the surface of T cells. Checkpoint blockade therapies release the inhibitory mechanism that control T-cell mediated immunity. The immune checkpoints are inhibitory pathways of immune cell that are important to regulate immune response and maintaining self-tolerance.

Once T cells are activated, they strengthen the immune system and boost an immune-mediated eradication of cancer cells (63). Immune checkpoints expressed on cytotoxic and regulatory T cells include programmed cell death protein-1 (PD-1 or CD279) and cytotoxic T lymphocyte associated antigen 4 (CTLA-4 or CD152) (64, 65) that interact with ligands cluster differential 80 (CD80), cluster differential 86 (CD86) and programmed death ligand-1 (PDL-1) on antigen presenting cells (APCs). ICIs prevent receptors and ligands from binding to each other, interrupting signals. In line with these considerations, the host immune system provides a powerful therapeutic target, thanks to its ability to precisely focus on tumor cells (66).

Despite the abovementioned advantages of immunotherapy, patients respond to ICIs heterogeneously and with a short-term efficacy (67). The reason why some tumors lack response is still unclear, although it probably depends on antigenicity and adjuvanticity defects, which are key factors in shaping the immunogenicity of tumor cells (68). Despite the fact that several biomarkers (PD-L1 expression, tumor-infiltrating lymphocytes, mutational burden, immune gene signatures and microsatellite instability) have been proposed, their sensibility and sensitivity are limited (69). Given that tumors with a high number of somatic mutations are more responsive to immunotherapies than the ones with a lower rate, the level of somatic mutations seems to be a crucial factor (70).

Preliminary data indicate that enteric microbiota may affect the efficiency of immunotherapy (71). It is well known that gut microbiota can modulate the peripheral immune system and that its diversity plays a crucial role in the maturation, development and function of both the innate and the adaptive immune systems (66, 72). Given the crosstalk between gut microbiota and immunity and considering that T cell infiltration of solid tumors, such as metastatic melanoma, is associated with favorable outcomes (73), microbiota could be considered as an important modulator of response to immunotherapy.

Along these lines, remarkable studies have demonstrated how the gut microbiota and its composition play a major role in the response to immunotherapy with ICIs, targeting the PD-1 and the CTLA-4 (74, 75).

With regards to the influence of gut microbiota on therapies targeting the PD-1/PD-L1 axis, Sivan et al. have provided important insights from murine models in 2015 (74). Indeed, they have demonstrated how genetically similar mice with different microbiota composition exhibited significant immune-mediated differences in melanoma growth rate. The intratumoral CD8+ T cell accumulation was found to be significantly lower in mice with a more aggressive tumor growth and a remarkable reduction in the difference of antitumor immunity was shown after cohousing, suggesting an environmental influence. Moreover, fecal suspensions derived from mice with less aggressive tumor growth were able to delay tumor growth and to enhance the induction and infiltration of tumor-specific CD8+ T cells in the other group of mice, thus supporting a microbe-derived effect. Microbiota composition could also influence the response to immunotherapy with antibodies targeting PD-L1. These abovementioned data support the idea that microbiota might be a source of intersubjective heterogeneity regarding spontaneous antitumor immunity and therapeutic effects of antibodies targeting the PD-1/PD-L1 axis.

A related research revealed how the antitumor effects of CTLA-4 blockade depend on distinct *Bacteroides* species, with a lack of response to CTLA-4 blockade in antibiotic-treated or germ-free mice (75). The analysis of microbiota composition showed *Bifidobacterium* being positively associated with antitumor T cell responses. Furthermore, *Bifidobacterium*-treated mice showed better tumor surveillance compared to their non-*Bifidobacterium* treated counterparts, together with a high increase of tumor-specific T cells in the periphery and a significant increase of antigen-specific CD8+ T cells within the tumor (74).

On the other hand, the treatment itself may affect microbiota composition. Indeed, in patients with metastatic melanoma, Ipilimumab can alter the abundance of gut *Bacteroides* spp. with an immunogenic power, especially *B. thetaiotaomicron* and *B. fragilis*, which, in turn, can affect its therapeutic effect. Feces rich in *B. fragilis* (except *B. distasonis* or *B. uniformis*) were negatively associated with tumor dimension after the therapy. Hence, the efficacy of CTLA-4 blockade is influenced by the microbiota composition (75). The gut microbiome and antibiotic therapies appear to impact the response to adoptive cell therapies in murine models (76, 77) and preliminary studies on haematological and solid tumor case series seem to align with this data (78).

Recent studies on humans have reported an unexpected role of specific members of the gut microbiota as predictors of response to immunotherapy in a distinctive series of epithelial tumors (NSCLC, renal cell carcinoma, and urothelial carcinoma) and melanoma patients (79–81). Routy et al. recently demonstrated how patients with epithelial tumors that responded to PD-1 blockade had differential composition of gut bacteria, being enriched in *Akkermansia* and *Alistipes*. Moreover, by performing a fecal microbial transplantation in mice it was demonstrated how there were enhanced responses related to the responders’ fecal material. In addition, the efficacy of anti-PD-1 in GF mice receiving non-responders’ transplantation could be restored by the administration of *Akkermansia muciniphila* alone or in combination with *Enterococcus hirae* (79). Regarding metastatic melanoma, a study by Gopalakrishan et al. revealed that responders to anti-PD-1 therapy not only had a significantly higher diversity of bacteria in their gut microbiota, but also had a higher relative abundance of *Clostridiales, Ruminococcaceae*, and *Faecalibacterium* spp. On the other hand, non-responders had significantly lower diversity of gut bacteria and a higher abundance of *Bacteroidales*. The composition of microbiota was related to the expression of cytotoxic T cell markers and the mechanism of antigen processing and presentation, which was increased in the first group of patients (80). In addition, another study has shown how the transplantation of stool to germ-free mice could improve the efficacy of anti-PD-L1 immunotherapy in mice that received responder-stool by increasing the density of CD8+ T-cells and reducing FoxP3+ CD4+ Tregs in the tumor microenvironment.

Given the recent findings of the microbiota being a significant modulator of response to ICIs, important insights are provided into the possibility of intervening on the composition of the intestinal microbiota to affect the ability to modulate antitumor immune responses. The crosstalk between microbiota and the immune system may allow a microbiota-based selection of patients that might benefit from a specific immunotherapy treatment, boosting their anticancer response. The prospect of being able to manipulate gut microbiota in order to modify the response to checkpoint inhibitors, serves as a continuous stimulus future research.

### The Microbiota Modulation of Drug Resistance

Besides regulating the response to checkpoint blockade therapies, gut microbiota can also take part in resistance to this kind of treatment, crowding out its therapeutic benefits. Xiaochang Xue et al. indicated that commensal bacteria act in a direct way on our immune cells, down-regulating the intestinal miR-10a expression. As they have shown, *E. coli* and flagellated A4 commensal bacteria manage to recognize and engage TLR1/2, TLR4, TLR5, TLR9 and NOD2 on dendritic cells (DCs), resulting in a down-regulation of miR-10a *via* the MyD88-dependent pathway (82). Considering that miR-10a inhibits DC production of IL-12/IL-23p40, miR-10a itself acts as a negative regulator of both innate and adaptive immune responses to microbiota (82). It is known that IL-12/IL-23p40 gene has a key role in the stimulation of Th1 cell-mediated immune responses and cytotoxic activity of CD8+ T and natural killer cells (83). Thus, their absence threatens the effectiveness of the anticancer immune response.

Furthermore, both Gram-positive and Gram-negative bacteria are able to produce extracellular vesicles (EVs), which carry carbohydrates, signaling molecules, metabolites, proteins, DNA, RNA, in order to create a cell-to-cell communication through the transport of their content (84). Bacterial EVs contain short RNAs (85) (sRNAs) and miRNA-sized sRNAs (msRNAs) (86), which have regulatory functions as well as miRNA in eukaryotic cells. Different studies (87, 88) confirm that the exchange of information between bacterial EVs and host cells through the modulation of the gene expression, might be involved in inducing resistance to chemotherapy and immunotherapy. On the other hand, even human intestinal epithelial cells release miRNAs encapsulated in EVs, which, as it has been demonstrated by S. Liu et al., may promote the growth *F. nucleatus* and *E. coli*, in order to maintain a physiological balance of our intestinal microbiota (89).

In conclusion, it is clear that there is a mutual influence between bacteria and human host cells, thus, it is conceivable that further studies could provide additional findings to better understand EV-mediated inter-cell communication and, perhaps, a new opportunity to reduce the resistance to cancer therapies by using specific probiotics, antibiotics or focusing on the composition of microbiome to personalize therapies.

## The Impact of Food on Gut Microbiota

### Diet

The contribution of diet to the modulation of microbiota and its crucial role in orchestrating the host–microbiota crosstalk is evident since the beginning of a human life when there is a microbiota-dependent relationship between milk oligosaccharides and growth promotion (90). This crosstalk between diet and microbiota continues and becomes more complex with the increased bacterial richness associated with the introduction of solid foods (91), and keeps affecting our lives until the end, with a decreased richness in the microbiota of frail ageing populations living in long-stay care, probably due to reduced food diversity (92).

A study demonstrated how the gut microbiome can respond to dietary interventions in humans in a rapid, diet-specific manner and how a diet composed entirely of animal products is able to trigger enrichment in bile-tolerant bacteria (*Alistipes, Bilophila* and *Bacteroides*) and depletion in *Firmicutes* that metabolize plant polysaccharides (*Roseburia, Eubacterium rectale* and *Ruminococcus bromii*) (93). Some more metagenomic and metabolomic analyses confirmed this trade-off between protein fermentation and degradation in protein-rich, animal-based diets, as opposed to carbohydrate fermentation and amino acid biosynthesis in plant-based diets (94). For example, the elimination of animal fats in the human diet was associated with a decrease in harmful *Bacteroidales* bacteria (95).

One of the dietary components that has shown to have a significant impact on the microbiota’s composition is fiber. Indeed, taking into consideration the different diet styles, it was shown how administering to mice a typical Western-style diet, that contains a relatively lower amount of fiber, could reduce the amount of *Bifidobacterium* and the gut microbiota diversity, leading to increased penetrability, and a reduced production rate of the inner mucus layer (96). Another study in healthy human volunteers (97), showed how the reduction in the amount of fiber intake led to a statistically significant reduction in the abundance of *Faecalibacterium prausnitzii* and *Roseburia* spp, which were positively correlated with the proportion of butyrate during both baseline normal diets. Moreover, a chronic lack of dietary fiber intake could lead to a reduced diversity in the gut microbiota (98). Preliminary data suggest that diet fiber intake could even impact the likelihood of response to anti-PD-1 treatment (99), providing interesting insights into the possible role of diet in the response to cancer therapies.

Many other dietary nutrients were studied for their roles in the modulation of gut microbiota, for example major groups of polyphenols assayed in both *in vitro* and preclinical studies have shown their ability to modulate the gut microbiota to a beneficial pool characterized by the abundance of *Bifidobacterium, Lactobacillus, Akkermansia*, and *Faecalibacterium* sp (100). Resveratrol is a naturally occurring polyphenol produced by some dietary botanicals, including red grapes (101), as a self-defence agent. Together with its cardio-protective and neuro-protective properties, it also serves as an antitumoral agent (102) which has shown the ability to induce antioxidant enzymes that attenuate oxidative stress (103).

Given the importance of these bacteria and their implications in cancer therapy, it is possible that diet could improve the patients’ outcomes through the modulation of their microbiome. Furthermore, considering that diet interacts with the human ‘holobiont’ in a person-specific way, being able to obtain multiple parameters from the host and its resident microbiota could assist in devising precision dietary interventions (104). This would provide a safe and simple opportunity for assessing the implication of microbiota and downstream immune manipulation in cancer patient populations.

Ongoing trials are currently exploring the impact that diet could have on the gut microbiota of oncologic patients. A randomized clinical trial that started in 2013 (NCT02079662) is currently studying how an integrative oncological program, that aims to make changes in the patients’ lifestyles and behaviors, including dietary recommendations and meal delivery, could influence long-term treatment results in patients with stage III breast cancer initiating radiotherapy. Interestingly, longitudinal gut and oral microbiome samples, along with a battery of questionnaires, are listed as secondary outcomes in order to better gauge how the microbiome might change in relation to behavioral patterns in cancer patients. A second trial (105) was designed to investigate fiber supplementation in patients with a previous history of colorectal cancer, through supplementation of beans into the normal diet for 8 weeks, to measure shifts in bacterial populations after a diet alteration. Even though both studies are not finalized yet, they will provide valuable information on how lifestyle factors can modulate the gut microbiome and its interaction with diet. A better understanding of the impact that diet has on microbiota will likely be key to the future of clinical and public health approaches to cancer.

### Probiotics

Despite the impact of dietary nutrients seems relatively simple and fast to design, it may be hard to monitor the patient’s compliance in dietary description intake; the effect of food on the microbiota might be modest and heavily host related. An alternative method that could provide much more control towards microbial manipulation could be the administration of probiotics.

Probiotics are living microorganisms that, when balanced in terms of quantity, grant beneficial effects to the host (106). It is well-established that probiotics act in different ways to prevent the colonization of pathogens, such as *Clostridium difficile* and *Staphylococcus aureus*, and, consequently, dysbiosis (107). Indeed, probiotics antagonize pathogen colonization by competing for nutrients (108), sticking to the epithelial cell surfaces or to the mucus (109) and creating clusters with pathogens themselves (110). They also have a role in producing metabolites, such as lactic acid, acetic acid and bacteriocins, which are able to lower luminal pH (111) and unleash a direct antimicrobial activity (112), in order to inhibit pathogen growth.

There has been an increasing interest towards probiotics potential role in improving antitumor immunity, considering their ability to repress colonic inflammation and to stimulate immunosurveillance (113).

*Bifidobacterium* and *Lactobacillus* are two of the most active probiotics, which have been identified as regulators of gut homeostasis (114, 115). Moreover, other probiotics improve gut barrier function, by restoring epithelial integrity (116). An innovative approach could consist of administering probiotics before, during, or after potentially “microbiota-disrupting” or “microbiota-modulated” treatments. There have been several clinical trials administering probiotics in CRC patients. One that was completed in 2017 (117), aimed to unveil the change in fecal and tumor microbiota from the baseline, after using probiotics containing strains of *L. acidophilus* and *B. lactis*. The results showed an increased abundance of butyrate-producing bacteria (above all *Faecalibacterium* and other *Clostridiales*) within the tumor, and its associated non-tumor colonic mucosa and stool. This is a demonstration that probiotic therapy can change colonic mucosa. Some other ongoing trials are assessing the impact of probiotic therapy on different types of cancer, including the change on CD8+ T cell infiltrate in patients with stage I-III breast cancer (NCT03358511), and thus, providing a perspective for a future better understanding of their influence on microbiome.

Nevertheless, even though probiotics are deemed safe and well-tolerated by healthy subjects, in patients with damaged intestinal barrier or compromised immunity, such as cancer patients, their physiological protection may fail (118), resulting in bacteremia, fungemia, endocarditis, liver abscess and pneumonia (119). In fact, many of the ongoing trials mentioned before, have focused on safety endpoints. There is definitely wide variability regarding the stability and composition of the available probiotic therapies’ formulations (120), and despite caution should be taken towards their use in cancer patients, the use of probiotics is not absolutely forbidden (113).

### Prebiotics

Prebiotics, introduced by Gibson and Roberfroid in 1995, are non-viable food components, which can stimulate the growth and the activity of specific gut bacteria, improving the host’s health (121).

Probiotics produce some kinds of prebiotics, such as short-chain fatty acids (SCFAs) (122). SCFAs are indeed produced by several bacteria in the gut that ferment fibers. Many SCFAs, such as acetate, butyrate and propionate, are important in maintaining intestinal homeostasis (123). Because of their ubiquitous presence, they are being studied for their potential as universal metabolic regulators of the immune system. Among them, it has been noticed that butyrate has a relevant role in CRC patients, inducing the apoptosis of cancer cells and inhibiting inflammation as well as oxidative stress (124). Though, it needs to be considered that every host has a different genetic background, which may interfere with butyrate beneficial effects (125).

Furthermore, prebiotic oligosaccharides with a low grade of polymerization may induce CD4+ T cells to produce IFN-γ and IL-10 (126). Besides, two different studies in which mice with a transplantable liver tumor have received inulin or oligofructose together with subtherapeutic doses of six chemotherapeutics, pointed out boosted chemotherapeutic effects and observed an increased lifespan (127, 128).

Despite the positive effects mentioned above, Singh et al. have also reported a harmful microbial fermentation as a result of prebiotic supplementation (129). Firstly, they tried to examine whether inulin has a mitigating effect towards metabolic syndrome in Toll-like receptor 5 (TLR5) knockout mice. Unfortunately, even though a long-term inulin enriched diet alleviates metabolic dysfunctions, concurrently, it promotes cholestasis and necroinflammation, and therefore it can induce hepatocellular carcinoma (HCC). However, a constant supplementation of inulin in drinking water revealed to trigger hepatic inflammation and fibrosis, but it did not promote tumor development. Additionally, similar effects have been induced by other soluble fiber, such as pectin and fructo-oligosaccharide, in contrast with some non-fermentable and insoluble fiber, such as cellulose, for instance. Interestingly, *Clostridia* species are highly present in mice which develop an HCC and a depletion in butyrate-producing bacteria has been reported to reduce the incidence of the hepatocellular carcinoma in TLR5 knockout mice (129).

In conclusion, the above submissions suggest that prebiotic fermentation and butyrate production have a partial contribution in the hepatocellular carcinoma development, although not being the decisive driver (113).

### Postbiotics

In addition to probiotics and prebiotics, an interesting role in the modulation of gut homeostasis and patients’ outcome is played by postbiotics, which are soluble products and metabolites derived from microorganisms (130). Instead of relying on bacteria supported by prebiotics or introduced through probiotics, postbiotics represent the microbial product itself, thus surpassing the bacteria (131). Despite the advantage of not being dependent on the cultivation of specific microbiota compositions, further characterization of postbiotic mechanism of action is still required.

In fact, it has been noted that *S. thermophilus* (132) and *E. coli* (133) generate supernatants, which protect rat gut from 5-FU-induced mucositis. In addition, p40, a soluble protein produced by *Lactobacillus rhamnosus* GG, avoids cytokine-induced epithelial apoptosis, prevents gut barrier dissolution (134, 135) and raises immunoglobulin A secretion (136). Moreover, an example of a molecule that can induce an immune phenotype in the absence of the microorganism is polysaccharide A (PSA) derived from *Bacterioides fragilis*. A study reported how this prominent human commensal can direct the conversion of CD4+ T cells into Foxp3+ Treg cells with the immunomodulatory molecule being polysaccharide A. Interestingly, polysaccharide A administration alone was sufficient to induce expansion of Tregs and to increase the production of anti-inflammatory IL-10 in mice *via* TLR2 activation. Furthermore, PSA was not only able to prevent, but also cure experimental colitis in animals (137). Despite microbial products are considered to be adjuvants stimulating the immune response, this study provides an insight into their ability to promote immune suppression as well.

Moreover, as mentioned before, SCFAs are gut microbiota-derived bacterial fermentation products that are being studied for their effect on the immune system. A study demonstrated how short-chain fatty acids regulate the size and function of the colonic Treg pool and protect against colitis in a Ffar2-dependent manner in mice (138). Another study showed that butyrate, produced by commensal microorganisms during starch fermentation, facilitated extrathymic generation of Treg cells and *de novo* Treg-cell generation in the periphery was potentiated by propionate (139).

In oncologic patients, postbiotics induce antitumor effects (140). In support of this possibility, a study published by Konishi et al. in 2016, showed that *Lactobacillus casei* ATCC334 supernatant contained a powerful tumor-suppressive molecule, identified as ferrichrome. Ferrichrome treatment could induce apoptosis through the activation of c-jun N-terminal kinase (JNK). Interestingly, despite the tumor-suppressive effect of ferrichrome on colon cancer cells was found to be greater than or equal to that of conventional CRC drugs, this postbiotic showed less of an effect on healthy intestinal cells (140).

Overall, these data demonstrate that exogenous bacterial metabolites mediate the communication between the commensal microbiota and the immune system and can be utilized to influence immune activity in order to maintain homeostasis and promote health.

**Figure 2:**
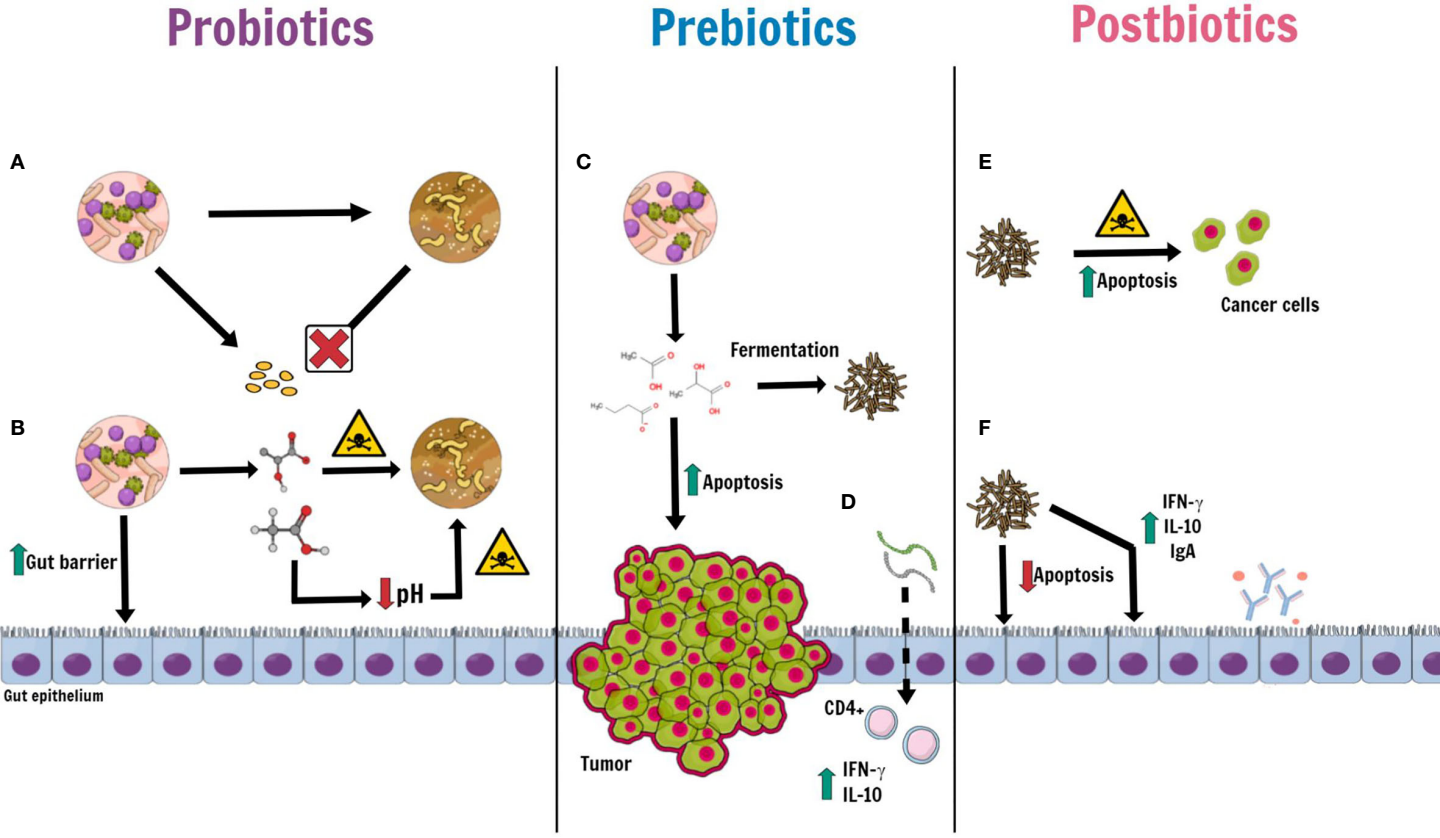
Putative mechanisms of actions of probiotics, prebiotics and postbiotics. Some kinds of probiotic, such as *Bifidubacterium, Lactobacillus, Faecalibacterium* and *Clostridiales*, may take an active role in maintaining gut homeostasis by: **(A)** preventing the proliferation and colonization of pathogens by competing for nutrients and microenvironment; **(B)** releasing antimicrobial peptides (such as lactic acid, acetic acid and bacteriocins) with a direct bactericidal effect and, indirectly, by lowering luminal pH. Moreover, probiotics induce an increase of mucin production, promote epithelial restoration and can enhance the expression of tight junctions. Prebiotics (inulin, oligofructose, soybean and oat fiber, pectin and non-digestible carbohydrates), derived from probiotics, **(C)** produce postbiotics through a fermentation process. Among prebiotics, Butyrate not only has an anti-inflammatory and antioxidative effect, but also an apoptotic effect against cancer cells, in CRC patients. **(D)** Oligosaccharides with a low grade of polymerization, directly absorbed by gut epithelium, stimulate T-cell CD4+ to release IFN-γ and IL-10. Postbiotics, prebiotics-derived products, on the one hand, **(E)** play a cytotoxic role against cancer cells, which increase their apoptosis; on the others **(F)** *Lactobacillus rhamnosus GG* and *Bacteriodes fragilis*, for instance, provide the wellness of the intestinal epithelium by inhibiting apoptosis of normal epithelial cells and raising the level of Ig A, IFN-γ and IL-10.

The putative mechanisms of actions of probiotics, prebiotics and postbiotics are shown in **Figure 2**.

### Antibiotics

Even though probiotics and prebiotics bring numerous modifications to the human gut microbiota, unluckily, all their benefits are transient (141–144). Evidence sustains that intestinal microbiota alterations, provided by antibiotics injection, result in an enduring loss of the original human microbiota diversity (145). Considering that patients’ response to immunotherapy partly depends on the varied composition of their microbiota, a loss in terms of abundance and types of microorganism species could affect therapeutic outcome.

A retrospective study investigated the negative association between the administration of antibiotics and ICIs. Patients that were recently given antibiotic therapy (ATB) had shorter Progression Free Survival (PFS) and Overall Survival (OS) when compared to those who did not receive ATB (146). Furthermore, the combination of ATBs and proton pump inhibitors has also been associated with gut dysbiosis, decreased bacterial richness, and the promotion of T-cell tolerance (147). It seems that antibiotic treatment might reduce the efficacy of ICIs by modifying the patient’s microbiota (80).

Ipilimumab is a wholly human monoclonal antibody against CTLA-4 that was approved in 2011 for the treatment of unresectable and metastatic melanoma, as well as adjuvant treatment for melanoma (148). It was found that patients on treatment with Ipilimumab developed antibodies against some elements of gut microbiota (149). On the other hand, a combination of broad-spectrum antibiotics, such as Ampicillin, Colistin and Streptomycin could compromise the antitumoral effects of CTLA–4–specific antibodies, suggesting that gut microbiota is crucial to set up the best anticancer treatment outcome through CTLA-4 blockade (75). Indeed, it has been shown that the administration of antibiotics interferes with the clinical benefit of anti-CTLA-4 therapy in mouse models and also PD-1-based immunotherapy both in mice and in humans (75, 79, 150). In a study involving a group of 74 patients with a stage IV melanoma, 10 of them received ATB 30 days prior to the administration of ICI, while the rest of the group has been treated with a single-agent ICI, among Pembrolizumab, Nivolumab and Ipilimumab, as first-line therapy. Patients of the ATB group had a PFS and an OS meaningfully shorter than those in the non-ATB group (151).

Another study examined the impact of broad-spectrum antibiotic treatments administered 1 month before the initiation ICI to 3 months thereafter, in patients with metastatic non-small cell lung cancer. Interestingly, a shorter duration of ATB did not impact patient prognosis when compared with a longer course, bringing light on the potential importance of the duration of antibiotic treatments (152). The abovementioned data suggest that the duration of broad-spectrum antibiotic treatments with respect to the initiation of ICI-based immunotherapy is important.

In conclusion, it needs to be considered that patients that need antibiotic therapies may have an enfeebled immune system and are therefore more likely to be subjected to bacterial infections and to be refractory to anticancer immunotherapy. Consequently, in order to reduce the negative impact of ATB on ICI treatments, it will be important to define the specific antibiotics that are more likely to negatively impact on the clinical outcome. Thus, using prebiotics and probiotics during ATB might be solicited to reduce the negative impact on microbiome composition induced by antibiotic therapy.

### Fecal Microbiome Transplantation

Fecal microbiota transplantation (FMT) represents the most direct way to affect microbiota, using complete normal human flora as a therapeutic probiotic mixture of living organisms. This type of bacteriotherapy has a longstanding history in animal health and is used against chronic infections of the bowel, including those infected by *Clostridium difficile* resistant to conventional therapies as well as other patient populations (153). Nonetheless, fecal microbiota transplantation is also one of the most used ways to prove that microbiota is able to upset the outcome of immunotherapy (74, 75, 80, 154–156).

**Figure 3:**
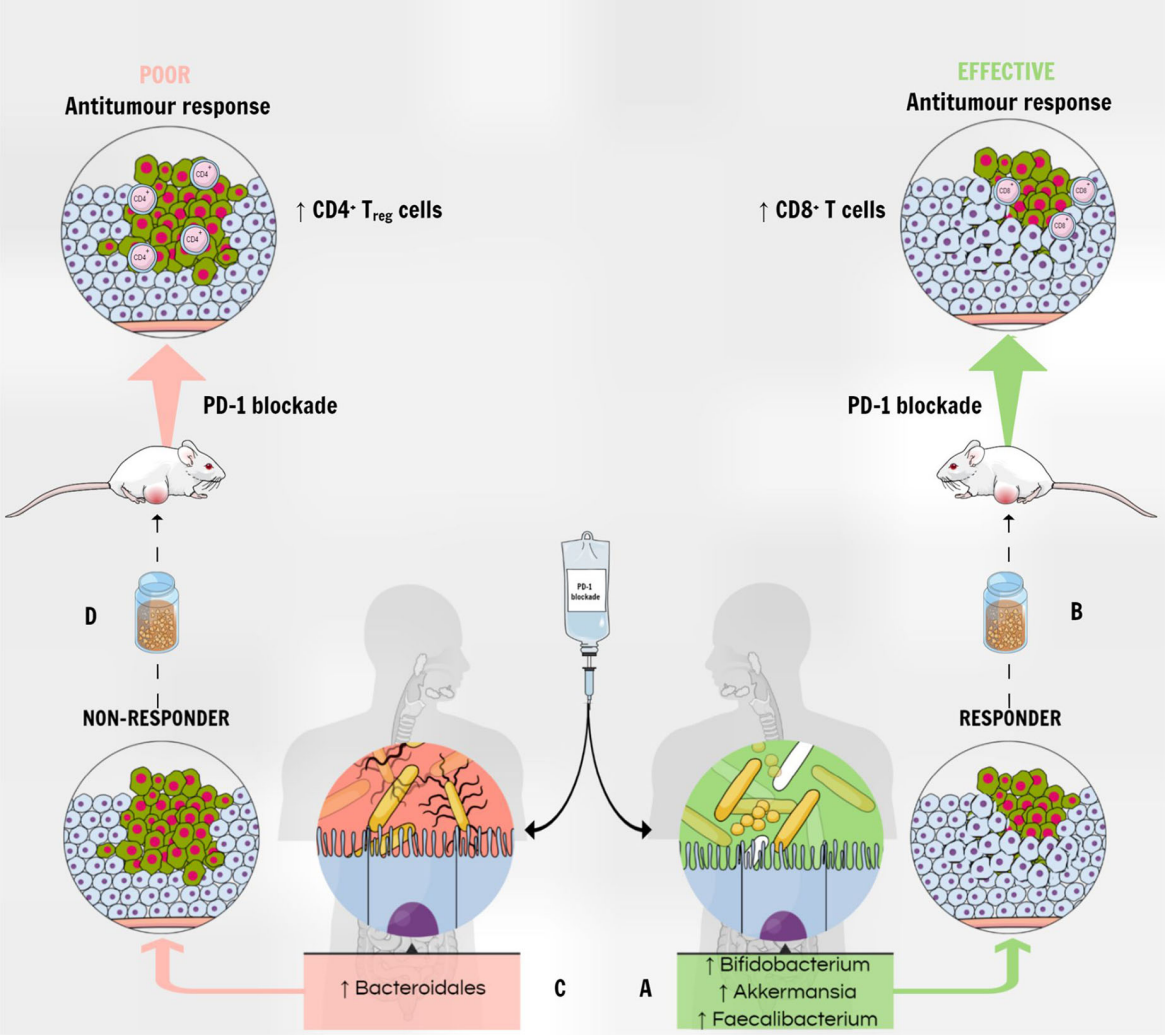
The gut microbiota modulates the response to PD-1 blockade therapy. **(A)** The enrichment of fecal microbiota with *Akkermansia muciniphila, Faecalibacterium* spp and *Bifidobacterium* spp correlates with a positive response to PD-1 immune-checkpoint blockade in patients with various types of tumors. **(B)** A fecal microbiota transplantation from responders into tumor-bearing mice correlates with increased antitumor CD8+ T cells in the tumor and improved response to anti–PD-1 therapy. **(C)** On the other hand, the higher abundance of *Bacteroidales* correlates with a deficient response to PD-1 blockade therapy in humans. **(D)** Mice receiving FMT from non-responders show poor anti-tumor response to anti–PD-1 therapy, and tumors show a higher density of immunosuppressive CD4+ T_reg_ cells.

Several studies aimed to show the impact of fecal microbiota transplantation in mice. Germ-free or antibiotic-treated mice that had received a fecal microbiota transplantation from patients who had a response to immune-checkpoint blockade, were enriched in CD45+ and CD8+ T cells, indeed correlating with a positive response to PD-1 immune-checkpoint blockade (80, 157) (**Figure 3**). On the other hand, fecal microbiota transplantation with feces from non-responders led to resistance to ICIs, with tumors having a high density of immunosuppressive CD4+ Treg cells (157).

Moreover, mice transplanted with feces from responders developed a higher response to anti-PD-L1 therapy (80, 154). It is noteworthy that, when fecal microbiota is enriched with *A. muciniphila*, as well as with *Faecalibacterium* spp and *Bifidobacterium* spp (80, 157), it correlates with a positive response to PD-1 immune-checkpoint blockade in patients with various types of tumors. Thus, *Bifidobacterium* in the gut is positively related to anti-tumor activity, especially by stimulating CD8+ T cells and DCs (60). In line with these observations, the use of antibiotics is related to lower clinical efficiency of immune-checkpoint blockade in different kinds of tumor tested in mice and patients (157).

Furthermore, clinical FMT trials are being considered in patients with both hematologic malignancies and solid tumors. The single-arm study “ODYSSEE” (158), explored the use of autologous fecal microbiota transplantation in acute myeloid leukemia patients treated with intensive chemotherapy and antibiotics. The aim was to restore the balance of their intestinal microbiome and thereby eradicate treatment-induced multidrug resistant bacteria, infection-related complications, as well as sequelae to the gastrointestinal tract. Moreover, in a Phase 1 clinical trial, FMT from patients that responded to immunotherapy is being administered to refractory patients with metastatic melanoma and unresectable stage III melanoma who failed at least one line of PD-1 blockade (159).

Recently, Baruch et al. reported the first-in-human clinical trials to test whether fecal microbiota transplantation can affect the response to anti–PD-1 immunotherapy in melanoma patients. In their phase 1 clinical trial, they investigated the safety and feasibility of FMT and the combination of FMT and reinduction of anti–PD-1 immunotherapy in 10 patients with anti–PD-1–refractory metastatic melanoma. They observed clinical responses in three patients, with FMT being associated with favorable changes in immune cell infiltrates and gene expression profiles in both the gut lamina propria and the tumor microenvironment (160). The design of new additional trials is currently underway, in order to test the hypothesis that the modulation of the gut microbiota can improve the response to treatment with ICIs (80).

These interesting preliminary findings offer compelling evidence for the ability of FMT to affect immunotherapy response in cancer patients, supporting the concept of overcoming resistance to immunotherapy by modulating the gut microbiota.

## Conclusions and Future Perspectives

The microbiome era has begun, and we have obtained substantial results on the influence of microbiota on cancer progression and treatment, including ICIs. The crosstalk between the host immune system and microbiota may allow a microbiota-based selection of patients that might benefit from a specific immunotherapy treatment, boosting their anticancer response. However, more studies on the topic are needed in order to better elucidate the microbial communities that colonize the tumor microenvironment, as well as the approaches to modulate the composition of gut microbiota.

Many dietary nutrients were studied for modulating gut microbiota, with fiber having shown a significant impact on the maintenance of microbiota diversity and the response to anti-PD-1 treatment. Since patients’ compliance might be hard to monitor and the effect of food on microbiota might be modest and heavily host related. An alternative method that could provide control towards gut homeostasis could be the use of prebiotic, postbiotic, probiotic and the administration of specific therapeutic schemes, for example with antibiotics. However, broader research is needed to determine the impact of these environmental factors on cancer therapy.

Satisfactory results offer compelling evidence on the ability of FMT to affect immunotherapy response in cancer patients. Further clinical trials with the use of FMT in cancer patients during ICIs are needed to better identify a strategy to overcome resistance to immunotherapy and improve patients’ outcomes.

Exploring the individual microbial profile and having a clear understanding of its interactions with various environmental factors could be a useful step to better modulate the gut microbiota. The prospect of being able to manipulate gut microbiota in order to modify the response to checkpoint inhibitors and set up personalized strategies serves as a continuous stimulus future research.

## Author Contributions

The authors contributed equally to this review. All authors contributed to the article and approved the submitted version.

## Conflict of Interest

The authors declare that the research was conducted in the absence of any commercial or financial relationships that could be construed as a potential conflict of interest.

## Publisher’s Note

All claims expressed in this article are solely those of the authors and do not necessarily represent those of their affiliated organizations, or those of the publisher, the editors and the reviewers. Any product that may be evaluated in this article, or claim that may be made by its manufacturer, is not guaranteed or endorsed by the publisher.
